# Molecular identification of *Sarcocystis lutrae* (Apicomplexa: Sarcocystidae) from the raccoon dog, *Nyctereutes procyonoides*, and the common raccoon, *Procyon lotor*, in the Czech Republic

**DOI:** 10.1186/s13071-020-04108-z

**Published:** 2020-05-06

**Authors:** Ondřej Máca

**Affiliations:** 1 Department of Pathology and Parasitology, State Veterinary Institute Prague, Sídlištní 136/24, Prague 6, 165 03 Czech Republic; 2 Department of Zoology and Fisheries, Faculty of Agrobiology, Food and Natural Resources, Czech University of Life Sciences Prague, Kamýcká 129, Prague Suchdol, 165 00 Czech Republic

**Keywords:** *Sarcocystis lutrae*, Common raccoon, Raccoon dog, Wildlife, Molecular characterization, Czech Republic

## Abstract

**Background:**

Apicomplexan parasites of the genus *Sarcocystis* have an obligate two-host life-cycle and comprise about 200 species, which infect different cold- and warm-blooded hosts, including humans. Recently, morphological and molecular studies of sarcocysts in broadly spread carnivore hosts have been on the rise. The description of muscular tissues infection by *Sarcocystis* in the raccoon dog and the common raccoon from the Czech Republic is herein presented.

**Methods:**

During January-August 2019, 15 raccoon dogs and 1 common raccoon were examined from 5 districts (Česká Lípa, Liberec, Mladá Boleslav, Trutnov and Ústí nad Labem) of the Czech Republic. Muscle parts (diaphragm, forearm, hind-limb, tongue and heart) were examined in wet preparations under compression by light microscopy. After finding *Sarcocystis* sp., morphological characteristics and molecular analyses of *18S* rRNA, *28S* rRNA, ITS1 and *cox*1 loci were used to identify the species.

**Results:**

Sarcocysts were detected and identified in 1 out of 15 raccoon dogs and in the single common raccoon. Preferential infection sites were diaphragm and tongue, followed by forearm and hind limb. To our knowledge, this is the first identification of microscopic sarcocysts by multi-locus genetic analysis from both host species. Molecular analyses revealed 100% similarity at *18S* rRNA, *28S* rRNA, and *cox*1 genes with *S*. *lutrae* for both hosts and 98–100% identity at the ITS1 region of the isolate from the common raccoon.

**Conclusions:**

Both widely distributed non-indigenous wild carnivores represent new intermediate host records for *S*. *lutrae* and the first report of this parasite in a member of the family Procyonidae, but still with an unknown natural definitive host. Molecular data revealed that this parasite species appears more closely related to the *Sarcocystis* spp. using raptorial birds as definitive hosts. Therefore, further studies aimed at its identification, including the complete life-cycle, remain necessary. 
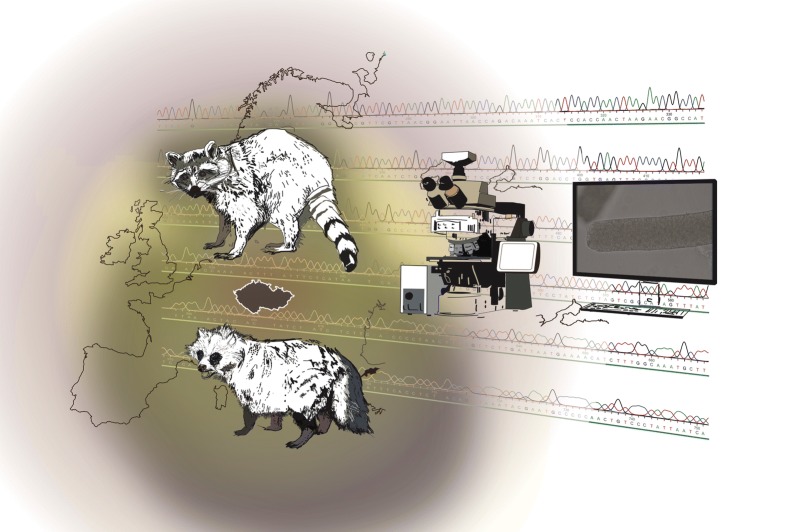

## Background

The genus *Sarcocystis* (Apicomplexa: Sarcocystidae) comprises approximately 200 species that are ranked among the most widespread protozoan infectious agents in domestic and wild animals, as well as humans, which act as intermediate or/and definitive hosts [1]. One of the most relevant *Sarcocystis* species in recent years is *Sarcocystis lutrae* that is recognised as a major cause of sarcosporidial infection in the skeletal muscles of carnivores, especially in mustelids (the American mink, *Neovison vison*; beech marten, *Martes foina*; European badger, *Meles meles*; European otter, *Lutra lutra*; European polecat, *Mustela putorius*) and less commonly in canids (Arctic fox, *Vulpes lagopus*; and red fox, *Vulpes vulpes*) [2–6].

The role of wild terrestrial carnivores in the transmission of infectious diseases continuously requires scientific attention, especially in those widely spread non-indigenous carnivores with the ability to adapt in new habitats, such as the raccoon dog (*Nyctereutes procyonoides*) (Canidae) and the common raccoon (*Procyon lotor*) (Procyonidae). These hosts are native to East Asia and North America and have been introduced across Europe, including the Czech Republic, where they have spread zoonotic parasites and other pathogens (e.g. *Echinococcus* or *Trichinella*) [7–10].

To date, *P*. *lotor* has been reported as an important natural intermediate host of *S. neurona* in North America [11], *S*. *kirkpatricki* in Illinois, USA [12] and *S.* cf. *sebeki* and two additional unnamed species in Germany [13]. On the other hand, *Sarcocystis* sp. was reported in the Japanese raccoon dog (*Nyctereutes procyonoides viverrinus*) from Japan [14], but never in *N*. *procyonoides ussuriensis*. In none of these reports the parasite has been described molecularly. Therefore, as a part of a study focusing on the evaluation of the diversity of *Sarcocystis* species in the Czech Republic, raccoon dogs and common raccoons were examined for the occurrence of parasitic infections. Moreover, parasites were for the first time molecularly characterized by using 4 loci, i.e. *18S* ribosomal RNA (*18S* rRNA), *28S* ribosomal RNA (*28S* rRNA), mitochondrial cytochrome *c* oxidase subunit 1 (*cox*1) genes and the internal transcribed spacer 1 (ITS1) region.

## Methods

Hunted or road-killed animals were sent to the State Veterinary Institute Prague as part of a monitoring programme during January-August 2019. Muscle samples were taken from 15 raccoon dogs from Česká Lípa, Liberec, Mladá Boleslav, Trutnov, as well as 1 common raccoon from Ústí nad Labem, all in the Czech Republic. Samples were immediately examined through wet mounts and compression methods (28 plots of compressors) for the detection of bradyzoites or sarcocysts of *Sarcocystis* using a Leica DMLB optical microscope (Leica Microsystems, Wetzlar, Germany) equipped with Nomarski differential interference contrast. Parasites were transferred to an Eppendorf tube for DNA extraction.

Genomic DNA was extracted by glass bead disruption from four isolates of sarcocysts collected from diaphragm or forearm muscles of both host species using the QIAamp^®^ DNA Stool Mini Kit (Qiagen, Hilden, Germany) following the standard protocol suggested by the manufacturer. DNA was stored at − 20 °C until use in polymerase chain reaction (PCR) assays. The identity of the isolated sarcocysts was assessed based on their morphological aspects and complemented by sequencing of *18S* rRNA, *28S* rRNA, ITS1 and *cox*1 loci. PCR reactions were performed and amplified using primers for the *18S* rRNA gene (Fext/Rext; Fint/Rint) [15], *28S* rRNA gene (KL1/LS2R; LS1F/KL3) [16, 17], ITS1 region (18S14F/ITS1FR or SU1F/5.8SR2) [17, 18] and *cox*1 gene (SF1/SR10 or SF1/SR5) [19, 20]. Amplification was carried out in a final volume of 25 μl (20 μl of reaction mixture and 5 μl of DNA extract) comprising of 1× Green or Colorless GoTaq^®^ Flexi Buffer, 2.5 mM of MgCl_2_, 0.625 U of GoTaq^®^ G2 Flexi DNA Polymerase (Promega, Madison, Wisconsin, USA), 0.2 mM of dNTP mix (Bioline, London, UK), 0.4 μM of each primer, DNA template and nuclease-free water. PCR amplification of isolated DNA samples plus positive and negative controls were performed with the cycling conditions as follows: an initial denaturation step at 95 °C for 5 min; 35 cycles of 95 °C for 30 s, 43.5–55.5 °C for 30 s, 72 °C for 1 min; and a final extension step at 72 °C for 10 min. The nested PCR (*18S* and *28S* rRNA genes) cycling conditions were identical to those used for PCR (ITS1 region and *cox*1 gene) amplification. The PCR products were later analysed by electrophoresis in a 1% (w/v) agarose gel stained with ethidium bromide and visualized under a UV light. The PCR products were purified using the High Pure PCR Product Purification Kit (Roche Diagnostics, Mannheim, Germany) and sequenced using the sequencing service of Eurofins Genomics (Ebersberg, Germany) from both forward and reverse directions for each isolate. The nucleotide sequences of the four loci derived in this study have been deposited in the GenBank database under the accession numbers MT036248-MT036254. The newly generated sequences were compared with published sequences across the GenBank NCBI database using BLAST analysis (Basic Local Alignment Search Tool).

## Results

**Fig. 1:**
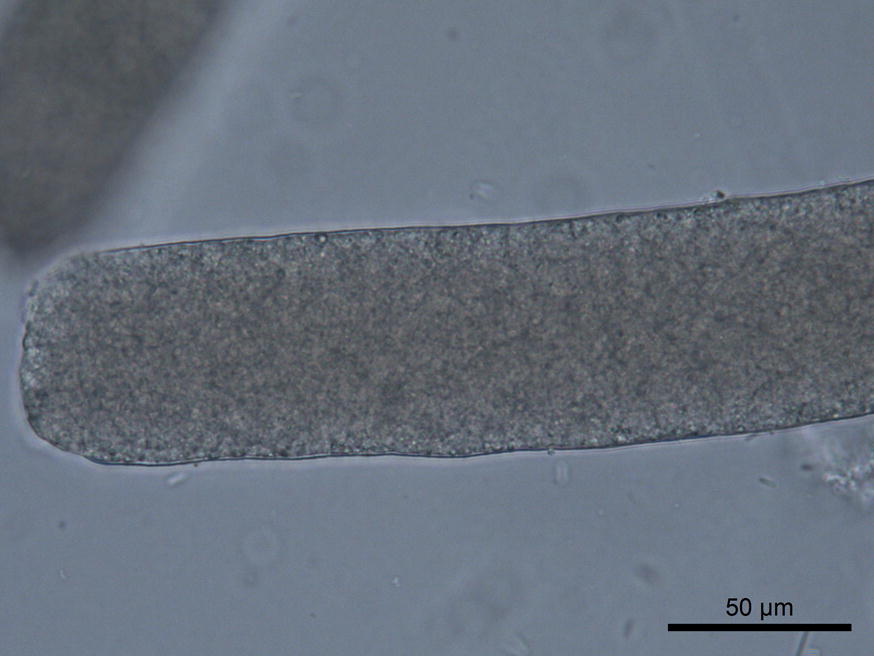
*Sarcocystis lutrae* isolated from the hind-limb muscle of *Nyctereutes procyonoides* (wet mount)

One sarcocyst morphotype (Fig. 1) and several bradyzoites of the genus *Sarcocystis* were detected in the diaphragm, hind-limb, forearm and tongue muscles in 1 of 15 raccoon dogs (prevalence = 7%; intensity = 1 sarcocyst per gram) and in the single common raccoon examined (100%, intensity = 1 sarcocyst per gram). Hearts of both hosts were negative. Sarcocysts were up to 2260 μm long, thin-walled (0.8–1.1 μm thick) and 66 μm wide, with a smooth wall and no visible protrusions. Sarcocysts were filled with banana-shaped bradyzoites, measuring 6.8–9.2 × 1.6–2.4 μm (*n *= 20).

Muscular sarcocysts from *N*. *procyonoides* (one isolate) and *P*. *lotor* (two isolates) were successfully identified molecularly and sequenced at each locus. In the former, the length of the generated representative nucleotide sequences was 1593 bp for *18S* rRNA, 519 bp for *28S* rRNA and 880 bp for *cox*1, whereas in the latter the length was 1525 bp for *18S* rRNA, 1279 bp for *28S* rRNA, 337 bp for the ITS1 region and 1029 bp for *cox*1. Sequence analysis at four genetic loci identified sarcocysts isolates as *S*. *lutrae*. Isolated sarcocysts from both host species showed 100% similarity. At the *18S* rRNA and *cox*1 genes, the new sequences were 100% identical to those of *S*. *lutrae* from *M*. *meles* (Mustelidae) in the Czech Republic (GenBank: MG372102, MG372106) and Lithuania (GenBank: MG272295, MG273670). The new *cox*1 sequences were also 100% identical to those of *S*. *lari* (GenBank: MF946584) from *Haliaeetus albicilla* (Accipitridae) in Norway. At the *28S* rRNA gene, the new sequences obtained from *N*. *procyonoides* (519 bp) and *P*. *lotor* (1279 bp) were both 100% identical to those of *S*. *lutrae* from *M*. *meles* and *L*. *lutra* in the Czech Republic (GenBank: MG372104, MG372105), whereas the longer sequence from *P*. *lotor* differed by a single nucleotide polymorphism at position 891 (from G to A) from sequences obtained from *M*. *meles* and *L*. *lutra* in Lithuania (GenBank: MG272285, MG272276). A sequence of the highly variable genomic ITS1 region of *S*. *lutrae* obtained from *P*. *lotor* was 98–100% similar to the sequences obtained from *M*. *meles* in the Czech Republic (GenBank: MG372108) and Lithuania (GenBank: MG272304). Unfortunately, the ITS1 region of the isolate from *N*. *procyonoides* could not be successfully sequenced, even upon re-sequencing.

## Discussion

To the best of our knowledge, this is the first worldwide report of *S*. *lutrae* parasitizing *N*. *procyonoides* and *P*. *lotor*, thus increasing its host spectrum to nine species. Sarcocysts of *S*. *lutrae* were previously reported and molecularly characterized in different species of mustelids and canids from the Czech Republic, Latvia, Lithuania, Norway and Scotland [2–6]. This parasite is the most common species among mustelids, with prevalence ranging between 67–82% in the European badger (*M*. *meles*) from Scotland and the Czech Republic [3, 5]. In the present survey its prevalence in *N*. *procyonoides* was low (7%), although this host probably acts as natural intermediate host, since other canids, such as red foxes (*V. vulpes*) from Latvia (0.2%) and Lithuania (2%) [4], also showed low prevalence. A similar pattern occurs in a congeneric species, *S*. *arctica*, in red fox (*V*. *vulpes*) (prevalence 3.8%) from the Czech Republic [21]. On the other hand, the single *P*. *lotor* examined was positive to *S*. *lutrae*, thus suggesting that the parasite occurs in various hosts, although the sample size should be increased. More data about prevalence and molecular analysis of *S*. *lutrae* in procyonids are needed to determinate its host spectrum and the role of these hosts in the life-cycle of this parasite.

Our finding of *S*. *lutrae* in hind-limb differs from that of Kirillova et al. [4], who found no sarcocysts of this parasite in 294 *N*. *procyonoides* from Latvia, even though they examined the same kind of muscle. These differences could be related to the availability of potential hosts and their interactions, regional parasite occurrence, low susceptibility of raccoon dogs to this parasite or to the quality of samples examined (fresh *vs* autolytic).

The present nucleotide sequences were identical to those of *S*. *lutrae* in the European badger and European otter from the Czech Republic, thus indicating that the parasite is spread across the country in various carnivore hosts, at the same time, with some mustelid and canid hosts from other European countries. In order to elucidate the real host spectrum of this parasite, it is important to molecularly characterize those records of *Sarcocystis* sp. in several intermediate [1] and other possible definitive hosts, since the morphology of sarcocysts and bradyzoites is rather similar among species. All published sequences of *S*. *lutrae* using the *18S* rRNA gene share high identity (> 99%) with those of *S*. *halieti* (GenBank: MF946587) and *S*. *lari* (GenBank: MF946588) in the white-tailed sea eagle (*H*. *albicilla*), *S. corvusi* (GenBank: JN256117) in the jackdaw (*Corvus monedula*) and *S. columbae* (GenBank: HM125054) in the wood pigeon (*Columba palumbus*), *cox*1 gene sequences were 100% identical to *S*. *lari* (GenBank: MF946584) and > 99% to *Sarcocystis* sp. in *Accipiter cooperii* (GenBank: KY348756), see also [4]. Therefore, other loci are recommended to distinguish closely related *Sarcocystis* species where carnivores are intermediate hosts, such as the *28S* rRNA gene and ITS1. *28S* rRNA gene sequences of *S*. *lutrae* were 98% or less similar to other closely related *Sarcocystis* species (e.g. *S*. *lari*), whose intermediate and definitive hosts are the great black-backed gull (*Larus marinus*) (GenBank: JQ733509) and white-tailed sea eagle (GenBank: MF946611), respectively. ITS1 showed strong support for the species delimitation [22], since our sequence was 86–92% similar to *S*. *lari* (e.g. GenBank: MF946597). Thus, *S*. *lutrae* appears most closely related to species that use medium-to-large size raptorial birds as definitive hosts. Differences in the known sequences of *S*. *lutrae* are a result of the intraspecific variability, especially within ITS1 [2, 4] and *28S* rRNA gene [5], as observed in the present loci from *P*. *lotor* (GenBank: MT036249 and MT036253). Despite some limitations (unsuccessful ITS1 sequencing), the data allowed to clearly determine that both mammal species are hosts of this parasite.

Thus, *P*. *lotor* is the first reported procyonid host for *S*. *lutrae*; this host and *N*. *procyonoides* act as intermediate hosts of this parasite.

## Conclusions

There are still gaps in the knowledge of the life-cycle of *S*. *lutrae*, although apparently, this species uses several carnivore hosts as intermediate and final hosts, which should be morphologically and molecularly characterized to provide new insights into the spread of this parasite species. Further studies considering predator communities may help determine their role in the introduction and transmission of disease into different wild and farmed populations.

## Data Availability

The sequences generated in the present study were submitted to the GenBank database under the accession numbers MT036248-MT036254.

## Abbreviations

cox1: Cytochrome c oxidase subunit 1
ITS1: Internal transcribed spacer 1
PCR: Polymerase chain reaction
rRNA: Ribosomal ribonucleic acid
